# Expression of an endosome-excluded Cd63 prevents axis elongation in *Xenopus*

**DOI:** 10.17912/micropub.biology.000334

**Published:** 2020-11-27

**Authors:** Jennifer Kreis, Ramona Bonß, Kerstin Feistel, Philipp Vick

**Affiliations:** 1 Institute of Biology, University of Hohenheim, 70599 Stuttgart, Germany

**Figure 1. Overexpressed Cd63 mutant does not localize to late endosomes but blocks axis elongation:**
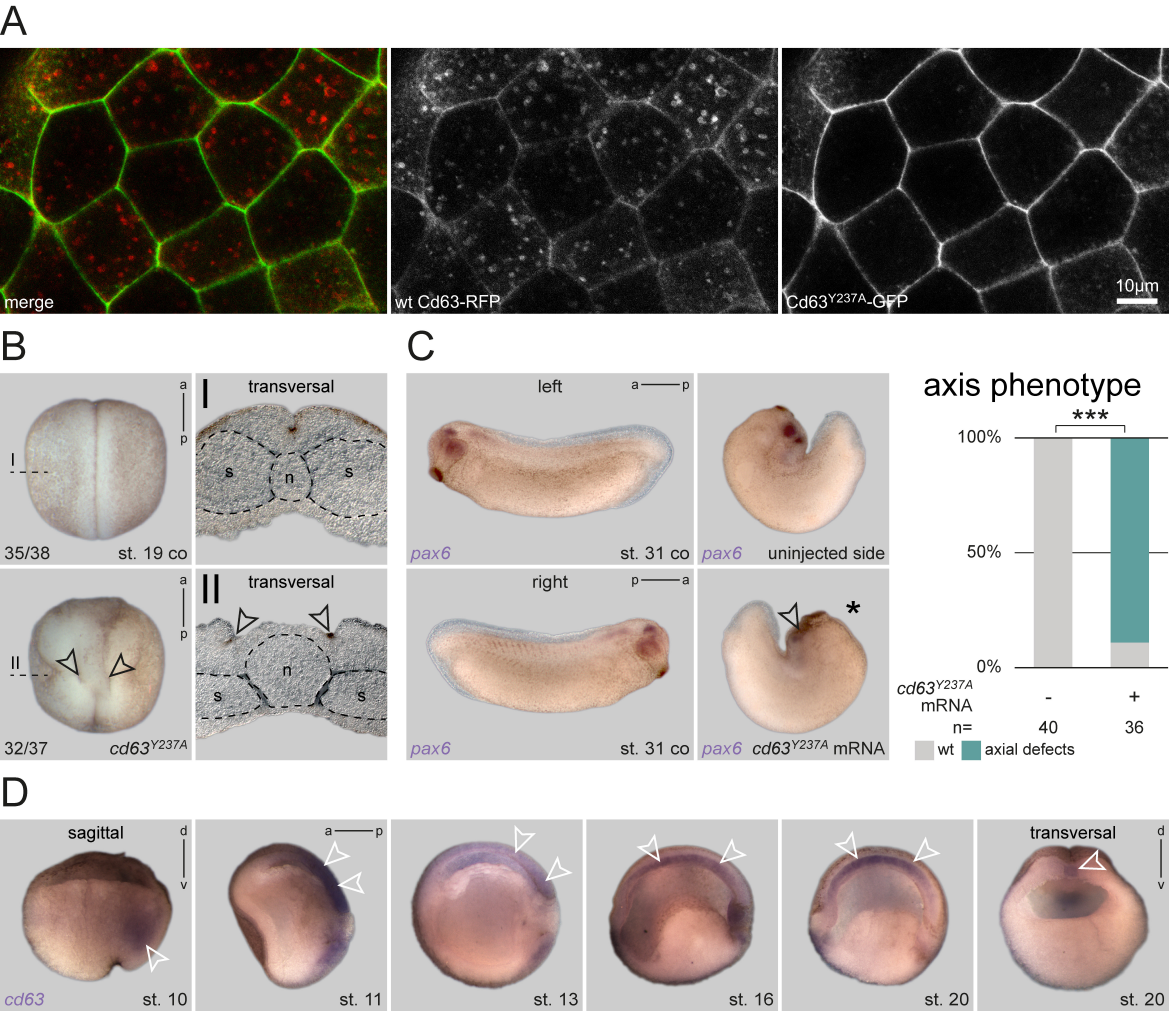
**A** Overexpressed RFP-tagged wt Cd63 (red) localizes to plasma membrane and endosomes in animal cap cells, whereas Cd63^Y237A ^(green) accumulates only in the plasma membrane. **B** Control specimen depicted normal neurulation and normal sized notochords (n, outlined by black dashed line) in transversal section. Overexpression of *cd63^Y237A^* mRNA in dorsal lineage caused defects in neural tube closure. Sections revealed strongly expanded notochordal tissue (outlined by black dashed line) and widened floorplate tissue, as indicated by apparently unaltered apical constriction at the hinge points lateral of it (arrowheads). **C** Untreated specimen of st. 31 established wt AP axis extension and wt expression of *pax6* indicating normal eye development. Unilateral overexpression (injected side marked by asterisk) of *cd63^Y237A^* mRNA resulted in embryos with shortened AP axis and reduced neural and optical expression of *pax6*. Quantification of axis phenotype. **D** Sagittal sections of st. 10 specimens revealed *cd63* transcripts in deep dorsal mesoderm (arrowhead). With ongoing gastrulation, expression was detected in the posterior neuroectoderm and notochordal mesoderm (st. 11; arrowheads). During neurulation (st. 13, 16, 20) *cd63* vanished from the neuroectoderm and became restricted to the notochord (arrowheads). Sagittal and transversal sections of late neurulas confirmed notochordal expression (arrowheads). a, anterior; AP, antero-posterior; co, control; d, dorsal; p, posterior; s, somites; st., stage; v, ventral; wt, wildtype.

## Description

Cd63 is an adapter protein belonging to the tetraspanin family. As other members, it possesses four membrane-spanning domains, two extracellular loops, and the N- and C-terminus both face the cytoplasm. Cd63 is highly enriched in intraluminal vesicles (ILV) of late endosomes (LE; also called multivesicular bodies, MVB; Termini and Gillette, 2017). Cd63 trafficking to LE occurs via classical routes, i.e. from the ER to the Golgi complex, then to the plasma membrane, from where it is endocytosed and incorporated into ILV (Kobayashi *et al.*, 2000; Pols and Klumperman, 2009). As an ILV-enriched component, Cd63 is a marker of exosomes, as these small extracellular vesicles are created from ILV of certain types of MVB (Pols and Klumperman, 2009; Simons and Raposo, 2009). Finally, Cd63 localizes to early melanosomes, highly specialized organelles that originate from LE-type precursors, and was shown to be required for melanogenesis in human melanoma cells (Basrur *et al.*, 2003; van Niel *et al.*, 2011). In our preceding analysis (Kreis *et al.*, 2020), using *Xenopus* embryos we found the orthologous gene expressed in the trunk neural crest (TNC) and in melanosome-associated tissues such as the retinal pigment epithelium and melanophores (pigment producing cells of poikilotherm vertebrates; Raposo and Marks, 2007). Neural knockdown of *cd63* specifically caused eye morphogenesis defects in early tadpole stages, surprisingly without impacting TNC formation and migration, nor melanophore specification and melanogenesis (Kreis *et al.*, 2020).

To analyze the cellular basis of this eye phenotype, we asked whether endosomal localization of Cd63 was required for correct eye development. Endocytosis and incorporation of Cd63 into ILV is mediated by a C-terminal ‘lysosomal targeting motif’, comprised of the last five amino acids (AA), GYEVM (Rous *et al.*, 2002). Single AA mutagenesis combined with indirect immunofluorescence screening had determined a tyrosine-to-alanine replacement (G**Y**EVM to G**A**EVM) to be most efficient in preventing translocation from the plasma membrane to LE in rat kidney cells (Rous *et al.*, 2002). We generated two equivalent constructs using *Xenopus* *cd63*: one comprising the mutated coding sequence (*cd63^Y237A^*/CS2+), and a second one fused N-terminally to eGFP (*cd63^Y237A^*/eGFP/CS2+) for localization testing. To verify that the *Xenopus* mutant construct mimicked the published mis-localization in embryos *in* *vivo*, we injected mRNA of *cd63^Y237A^*/eGFP together with an RFP-tagged wt *cd63* (Lee *et al.*, 2007) into the animal hemisphere and compared their subcellular localization by confocal microscopy. While WT Cd63 localized to both plasma membrane and endosomal structures as published (Lee *et al.*, 2007), mutant Cd63^Y237A^ was not found in endosomal structures, but was strongly enriched in the plasma membrane (Figure 1A). Thus, our construct provided a valuable tool to analyze the function of Cd63 localization to LE during *Xenopus* development.

Next, we injected the construct into the dorsal lineage of 4-cell stage embryos to analyze potential phenotypes. Surprisingly, injected embryos developed strong axial phenotypes during neurulation, resulting in an open neural tube at late neurula stages. Transversal sections of such specimens at the trunk level revealed that bilateral hinge points elevating the neural folds had formed correctly. However, the floorplate between these hinge points was extremely wide, preventing neural fold apposition and fusion (Figure 1B). Additionally, the notochord appeared amorphic in these specimens, not displaying a regular round shape as in control embryos, i.e. notochord morphogenesis was severely compromised. Together, the observed phenotype indicated a failure of proper axial elongation. This phenotype became even more pronounced, when injected embryos were reared to tailbud stages. Even in unilaterally injected specimens, axial extension of dorsal tissues was significantly inhibited, resulting in an extreme dorsal curvature in most embryos (Figure 1C). When such embryos were processed for *in situ* hybridization (ISH) to highlight eye development using a *pax6* probe, optic *pax6* expression was strongly reduced on the injected side (Figure 1C), indicating impairment of eye morphogenesis, and reminiscent of the morpholino (MO)-mediated knockdown of *cd63* (Kreis *et al.*, 2020).

As these phenotypes suggested a possible role of Cd63 in tissues important for axial convergent extension (CE) behavior (Keller and Sutherland, 2020), we next performed ISH to analyze the expression patterns of *cd63* during gastrulation and neurulation. *cd63* was expressed in both the axial mesoderm and the neuroectoderm (Figure 1D), i.e. germ layers exhibiting CE and extensive axial elongation. In bisected embryos, neural plate expression was visible during late gastrulation and early neurulation and vanished afterwards. Mesodermal expression was found continuously throughout this embryonic time-frame. *cd63* was first expressed in the deep mesoderm during early gastrulation, later, during formation and elongation of the notochord, these tissues showed intense mRNA enrichment as well. Together, these results support the conclusion that localization of Cd63 to LE is involved in regulation of embryonic axial extension during late gastrulation and/or early neurulation.

While the observed phenotype is striking, it also reveals a significant difference to our previous *cd63* loss-of-function approach (Kreis *et al.*, 2020). By inhibiting translation, we found *cd63* to be required for eye morphogenesis, with aberrant optic vesicle formation during late neurulation being the first visible phenotype. While such knockdown embryos did not show early axis elongation phenotypes, both approaches resulted in later eye defects. Therefore, the early defects presented here, indicate a dominant-inhibitory effect caused by plasma membrane retention of Cd63 (cf. Figure 1A). Therefore, the lack of endosomal trafficking of Cd63^Y237A^, which partially mimics the loss of *cd63*, could indicate a specific requirement for correct eye morphogenesis. In this work, we have not directly assessed if Cd63^Y237A ^mislocalized to the plasma membrane in the dorsal mesoderm or posterior neural ectoderm as well, i.e. in those tissues performing axis elongation behavior. However, if this was the case, as expected, this might also result in aberrant accumulation of Cd63 binding partners, which in turn caused Cd63^Y237A^-specific interference with CE behavior in these early stages.

Tetraspanins are well-known to serve as multipurpose adapters by forming ‘tetraspanin-enriched microdomains’. In this way, they have been reported to influence cell adhesion properties, and thus to alter cellular morphology and migratory behavior (Termini and Gillette, 2017). Supporting this notion, Cd63 is considered a negative driver of advanced stages of melanoma, as it inhibits epithelial-mesenchymal transition (EMT), and thus invasive migratory behavior (Radford *et al.*, 1997; Jang and Lee, 2003; Lupia *et al.*, 2014). The role of Cd63 in cell migration is also associated with its well-known regulatory function on integrin abundance at the membrane (Pols and Klumperman, 2009). In turn, correct integrin function and localization has been demonstrated to be required for CE movements in *Xenopus* (Marsden and DeSimone, 2003; Davidson *et al.*, 2006; Keller *et al.*, 2008). Thus, Cd63^Y237A ^might indirectly inhibit axial elongation and CE by negatively influencing integrin dynamics, potentially also in tissues normally not expressing *cd63* endogenously (i.e. in the neural plate during late neurula stages). In this context, it is important to mention that the observed phenotype (Figure 1B) is highly reminiscent of a CE defect as observed after blocking dishevelled (*dvl*) function specifically in the neural ectoderm. Upon *dvl* perturbation, neural folds also show apical constriction and medial movement at mid neurulation, but CE is not occurring in the midline in between (Wallingford and Harland, 2002). Dvl regulates CE in both floorplate and notochord by participating in the planar cell polarity pathway (PCP) via its PDZ protein interaction domain (Wallingford and Harland, 2001). Interestingly, Cd63 possesses a PDZ-binding motif in its C-terminus, which has been demonstrated to bind the PDZ domain of Syntenin-1 (Latysheva *et al.*, 2006; Pols and Klumperman, 2009). Syntenin-1, on the other hand, has been shown to interact with PDZ-binding domains of Syndecans, and Syndecan-4, a well-known regulator of PCP, is also involved in recycling of integrins, i.e. sharing a similar role with Cd63 (Grootjans *et al.*, 1997; Muñoz *et al.*, 2006; Morgan *et al.*, 2013). Future analyses will reveal whether there is a direct or indirect interaction of Cd63 with Dvl or Syntenin-1, which would also explain its effect on axial elongation during *Xenopus* neurulation.

## Methods

***Xenopus laevis* care and maintenance**

Frogs were purchased from Nasco (Fort Atkinson, WI, USA). Handling, care and experimental manipulations of animals was approved by the Regional Government Stuttgart, Germany (V349/18ZO ‘*Xenopus* Embryonen in der Forschung’), according to German regulations and laws (§6, article 1, sentence 2, nr. 4 of the animal protection act). To induce ovulation, female frogs were injected subcutaneously with 300-700 units of human chorionic gonadotropin (Sigma). Embryos were staged according to Nieuwkoop and Faber (1994). Only clutches of healthy embryos from healthy females were used for the experiments shown. Embryos from one batch were randomly picked and used as control or tested specimens.

**Construct cloning, mRNA synthesis and microinjections**

To generate an endosome-excluded version of *cd63*, the full-length *X. laevis* L-homeologwas isolated and the lysosomal targeting motif GYEVM was mutated to GAEVM by changing the codon TAT to GCT using the following primer pairs:

F1_5’-GAATTCCATGGCGGTGGAAGG-3’ (for both plasmids)

R1_5’-CTCGAGCGGTCACATGACTTCAGCTCCA-3’ (for CS2+ plasmid)

R2_5’-CTCGAGCGGTCCCATGACTTCAGCTCCA-3’ (for CS2+/c-terminal eGFP plasmid)

PCR products were introduced into CS2+ or CS2+/c-terminal eGFP plasmids using EcoRI and XhoI enzymes. For mRNA synthesis, plasmids were linearized with NotI and transcribed *in vitro* (Sp6 polymerase) using Ambion message machine kit. Drop size was calibrated to 4 nl per injection. 560pg (for double injections in B) or 400pg (for single injections in A, C) of mRNA was injected.

***In situ* Hybridization**

ISH probes for *X. laevis* *cd63* was used as described (Kreis *et al.*, 2020). For *in situ* mRNA detection, ISH was performed after fixation in MEMFA for 2-3 h at room temperature and processed following a standard protocol (Sive *et al.*, 2000). RNA *in situ* probes were transcribed using SP6 or T7 polymerases.

**Embryo sections**

For vibratome sections (thickness: 30-35 μm), embryos were embedded in a glutaraldehyde-crosslinked gelatin-albumin mix (embedding medium: 2.2 g gelatine, 135 g bovine serum albumin, 90 g sucrose dissolved in 450 ml PBS) and razor blade-sectioned as indicated in whole-mount panels using a Leica VT1000S vibratome.

**Photo Documentation**

Confocal images of fluorescence were taken with a Zeiss LSM 700 and adjusted using the Zeiss Zen 2012 Blue edition. Embryo pictures were taken with a Zeiss SteREO Discovery.V12 (whole embryos), or an Axioplan2 (sections) microscope using AxioVision 4.6. Adobe Photoshop CS6 was used for cropping and careful brightness adjustments. All figures were arranged using Adobe Illustrator CS6.

**Statistical analysis**

Statistical calculations were performed using Pearson’s chi-square test. *=p<0.05, **=p<0.01, ***=p<0.001 were used.
